# Pancreatic cancer cell/fibroblast co-culture induces M2 like macrophages that influence therapeutic response in a 3D model

**DOI:** 10.1371/journal.pone.0182039

**Published:** 2017-07-27

**Authors:** Janina Kuen, Diana Darowski, Tobias Kluge, Meher Majety

**Affiliations:** Discovery Oncology, Roche Innovation Center Munich, Roche Pharma Research and Early development, Penzberg, Germany; University of South Alabama Mitchell Cancer Institute, UNITED STATES

## Abstract

Pancreatic cancer (PC) remains one of the most challenging solid tumors to treat with a high unmet medical need as patients poorly respond to standard-of-care-therapies. Prominent desmoplastic reaction involving cancer-associated fibroblasts (CAFs) and the immune cells in the tumor microenvironment (TME) and their cross-talk play a significant role in tumor immune escape and progression. To identify the key cellular mechanisms induce an immunosuppressive tumor microenvironment, we established 3D co-culture model with pancreatic cancer cells, CAFs and monocytes. Using this model, we analyzed the influence of tumor cells and fibroblasts on monocytes and their immune suppressive phenotype. Phenotypic characterization of the monocytes after 3D co-culture with tumor/fibroblast spheroids was performed by analyzing the expression of defined cell surface markers and soluble factors. Functionality of these monocytes and their ability to influence T cell phenotype and proliferation was investigated. 3D co-culture of monocytes with pancreatic cancer cells and fibroblasts induced the production of immunosuppressive cytokines which are known to promote polarization of M2 like macrophages and myeloid derived suppressive cells (MDSCs). These co-culture spheroid polarized monocyte derived macrophages (MDMs) were poorly differentiated and had an M2 phenotype. The immunosuppressive function of these co-culture spheroids polarized MDMs was demonstrated by their ability to inhibit CD4+ and CD8+ T cell activation and proliferation in vitro, which we could partially reverse by 3D co-culture spheroid treatment with therapeutic molecules that are able to re-activated spheroid polarized MDMs or block immune suppressive factors such as Arginase-I.

## Introduction

Pancreatic ductal adenocarcinoma (PDAC) is one of the most lethal malignancies worldwide with an overall 5-year survival rate of only 7% [1]. Currently, there is no effective therapy not only due to the lack of screening methods to detect PDAC in early stages but also and because PDAC cells quickly acquire resistance to standard-of-care treatment such as combinations of chemo- and irradiation therapy [1]. One of the hallmarks of PDAC is a strong desmoplastic reaction largely involving tumor-associated fibroblasts, extracellular matrix (ECM) proteins, stellate cells as well as immune cells, which play a significant role in tumor progression and resistance to therapy [2, 3]. Infiltration and the phenotype of tumor infiltrating immune cells has been shown to predict clinical outcome in PDAC. Presence of tumor-associated macrophages (TAMs), in particular, has been shown to favour tumor progression, facilitating nodal lymph angiogenesis and promoting metastasis [4]. TAMs originate from circulating monocytes and show a high level of diversity and plasticity. Depending on the stimulus and the microenvironment, TAMs can phenotypically differentiate into either “alternatively activated” M2-macrophages with pro-tumorigenic properties driven by tumor derived environmental factors such as IL-10 and IL-4 or “classically activated” M1-macrophages, which are characterized by a pro-inflammatory phenotype, showing increased expression of HLA-DR and enhanced production of IL-12 and IL-6 [5, 6]. TAMs have not only been associated with angiogenesis, invasion and metastasis, but also with strong immunosuppressive properties. They are known to suppress T cell activation and proliferation by different mechanisms including secretion of immunosuppressive cytokines/factors such as TGF-β, IL-10 and type-I Arginase and by expressing cell surface molecules like PD-L1 that suppress T cell activation/function upon ligation [5, 7–9]. These complex interactions between various cell types within the tumor microenvironment direct the development of tumors *in vivo*. It is, however, challenging to mimic these interactions *in vitro*. Conventional 2D cell culture monolayers fail to optimally represent bi-directional communication between different cell types, ECM formation and the immunosuppressive milieu and are poor predictors of therapeutic drug testing [10]. To provide more physiologically relevant *in vitro* models, researchers turned to 3D cell culture systems to mimic *in vivo* tumor conditions by obtaining a 3D architecture that provides appropriate ECM proteins, cell-cell communication, nutrient gradients and hypoxic tumor regions [11, 12].

To this end, we previously established a 3D mono and co-culture model with lung, breast and pancreatic cancer cell lines and tumor-associated fibroblasts and identified soluble factors such as IL-6, CCL-2 and GM/M-CSF in co-culture supernatants that influence tumor growth and progression [13, 14]. We extended this tumor cell/fibroblast 3D co-culture model to study the influence on cell-cell contact and soluble immune modulators on monocyte differentiation and functionality. To our knowledge this is the first report showing the influence of PDAC cell/fibroblast 3D co-culture on monocyte differentiation *in vitro*. We also show that this 3D *in vitro* co-culture system reflects the *in vivo* situation and can be used as a tool to investigate the phenotypic changes induced due to co-culture.

## Material and methods

### Ethics statement

The study was approved by the local ethics committee (Bayerische Landesärztekammer, Munich) and subjects gave written informed consent.

### Cell lines and reagents

PaTu-8902, BxPc3, HPAC and MiaCaPa-2 tumor cell lines and MRC-5 foetal lung fibroblasts were maintained for passaging in cell culture tissue flasks in media containing 10% FCS, 2mM L-glutamine, 1% penicillin-streptomycin (Pen/Strep), 1% sodium pyruvate and 1% non-essential amino acids (NEAA) as recommended for each cell line by ATCC. For experiments, media was switched from culturing media to serum-free DMEM containing 5% Panexin NTA lacking hormones, insulin and growth factors, 2mM L-glutamine, 1% Pen/Strep and 1% NEAA. Cells used for further experiments were all below passage 16.

Mouse anti-human antibodies were purchased from BD Pharmingen^™^: CD206 APC (clone 19.2), HLA DR APC (clone G46-6), CD86 PE (clone Fun-1), CD80 FITC (clone L307.4), CD16 PE (clone 3G8), PD-L1 APC (cloneMIH1), CD25 PE-Cy7 (clone M-A251), CD69 BV510 (clone FN50), 4-1BB APC (clone 4B4-1). CTLA-4 BV421 (clone BNI3), PD-1 BV510 (clone EH12.1), mouse IgG1 APC (clone MOPC-21), mouse IgG1 PE (clone MOPC-21), mouse IgG2a APC (clone G155-178), mouse IgG1 FITC (clone MOPC-21), mouse IgG2a BV421 (clone G155-178), mouse IgG1 BV510 (clone X40), moues IgG1 PE-Cy (MOPC-21); from Biolegend: CD14 APC-Cy7 (clone M5E2), CD11b Pe-Cy7 (clone ICRF44), CD33 PerCP-Cy5.5 (clone WM53), mouse IgG1 PerCP-Cy5.5 (clone MOPC-21), mouse IgG2a APC-Cy7 (clone MOPC-173), CD40 FITC (clone 5C3); from R&D Systems: Arginase-1 APC (polyclonal sheep IgG), polyclonal sheep IgG APC; from Cell Signaling Technology^®^: EpCAM PE (clone VU1D9), EpCAM Alexa Fluor 647 (clone VU1D9), mouse IgG1 Alexa Fluor 647 (clone MOPC-21), mouse IgG1 PE (clone G3A1).

### 3D Co-cultures and cell viability assay

3D co-cultures were performed by coating 96 well U-bottom plates (Corning^®^ Costar^®^, #3799) and 6 well plates (Corning^®^ Costar^®^, #3516) with poly-2-hydroxyethyl methacrylate (poly-Hema) (#18894–100, Polysciences Europe GmbH). In poly-Hema coated 6 well plates, 2.5x10^5^ tumor cells were seeded per well as mono-culture and 1x10^5^ tumor cells and 1.5x10^5^ MRC-5 fibroblasts per well for co-cultures. For poly-Hema coated 96 well plates, 5000 tumor cells were seeded per well for mono-culture and 2000 tumor cells and 3000 MRC-5 fibroblasts per well for co-cultures. Monocultures and co-cultures were incubated for 5 days at 37°C in an incubator with 5% CO_2_ until spheroid formation.

Cell viability was measured using CellTiterGlo (Promega, #G7571) on day 5, 7, 9 and 11 according to the supplier’s instructions. Briefly, equal volume of CellTiterGlo reagent was added to the wells containing mono/co-cultures with and without monocytes in medium and incubated for 45min at room temperature (RT) on a shaker. The cell suspension was then transferred to a black 96 well clear flat bottom plate and the relative luminescence units (RLU) were measured using a microplate reader (Synergy 2 Plate reader, Bio-Tek). To compare spheroids with and without monocytes and to compensate monocyte numbers in this assay, we measured 10.000 freshly isolated monocytes alone as control and subtracted the relative luminescence unit (RLU) value from the spheroid RLU value with monocytes for each day.

### Measurement of secreted cytokines and growth factors

Supernatant from mono/co-cultures was collected on day 5 and day 11 of spheroid formation after addition of monocytes. Levels of 19 cytokines were determined by using Milliplex^®^ MAP Human Cytokine/Chemokine Panel (Millipore^™^) according to manufacturer´s instructions. Samples were run undiluted in duplicates. Briefly, standards, quality controls and samples were incubated in a 96 well plate with capture antibody-coated beads for two hours at RT under agitation. Beads were washed as recommended and detection was performed by biotinylated detection antibodies and streptavidin-PE conjugates. The fluorescence signals were determined on Luminex^®^ Bio-Rad 200 System; data were then analyzed using the Excel software.

### Monocyte isolation and *in vitro* generation of monocyte-derived macrophages (MDMs)

Monocytes were isolated by negative selection using the EasySep^®^ Human Monocyte Isolation Kit (Stemcell^™^ Technologies, #19359) according to manufacturer´s instructions. Shortly, blood from healthy donors was collected and PBMCs were obtained by a density gradient using Pancoll separation solution (Pan^™^ Biotech GmbH, #P04-60125) and washed 3 times with PBS, 2% FCS and 1mM ETDA. PBMCs were incubated with antibody isolation cocktail (50μl/ml) and platelet removal cocktail (50μl/ml) for 5 min followed by addition of magnetic beads. Negative selection of monocytes was then performed by removal of unwanted cells through magnetic separation of beads. For MDM differentiation, 1.2x10^6^ monocytes were seeded in DMEM + 5% Panexin NTA + 2mM L-glutamine with 1% Pen/Strep in a 6 well plate for 6 days in the presence of appropriate cytokines. For M1 macrophage polarization, monocytes were incubated with 100ng/ml rHuGM-CSF (Biolegend, #572905) for 3 days and then activated with 10ng/ml LPS (Imgenex, #2204) and 50ng/ml rHuIFN-y (Biolegend, #570206) for 3 additional days. For M2c macrophage polarization, monocytes were incubated with 100ng/ml rHuM-CSF (Biolegend, #574808) and 10ng/ml rHuIL-10 (Biolegend, #571004). Myeloid-derived suppressor cells (MDSCs) were obtained by incubation of monocytes with 100ng/ml rHuGM-CSF and 50ng/ml rHuIL-6 (Biolegend, #570804). Phenotypical and functional characterization was assessed after 6 days. For generation of spheroid-polarized MDMs, 1x10^4^ monocytes were added to co-culture spheroids per well on day 5 of spheroid formation. The co-cultures were incubated for 6 additional days without addition of polarizing cytokines.

### T cell suppression assay

T cells were obtained from freshly isolated PBMCs by negative selection using the EasySep^®^ Human T cell isolation kit (Stemcell^™^ Technologies, #17951) according to manufacturer´s instructions. Briefly, PBMCs were isolated from healthy blood donors and incubated with 50μl/ml antibody isolation cocktail for 5 min followed by addition of 40μl/ml magnetic rapid sphere beads. Negative selection of pan T cells was performed by removal of unwanted cells by magnetic separation of beads. T cells were then washed with PBS and labeled with 5μm Carboxyfluorescein succinimidyl ester (CFSE) for 5 min in the dark. On day 11, 5x10^4^ T cells were seeded per well in a poly-Hema coated 96 well plate already containing co-culture spheroids with and without spheroid-polarized MDMs. As proliferation controls, T cells were also incubated either alone or with monocytes. To activate T cells, anti-CD3 and anti-CD28 activation beads (Invitrogen^™^, #11131D) were washed twice and added to wells using a ratio of 1 bead per 16 T cells. In order to restore T cell proliferation, co-culture was treated with either 1mM Arginase-I inhibitor (nor-NOHA, MerckMillipore, #189302-40-7), 500μM iNOS inhibitor (1400W dihydrochloride, Tocris, #1415), 25ng/ml TLR8 ligand (TL8-506, Invivogen, #tlrl-tl8506), 250ng/ml human CD40 ligand (Miltenyi Biotec, #130-096-714) or Arginase-1/iNOS inhibitor and TLR8/CD40 ligand combinations one day prior T cell addition. Surface marker expression was analyzed and proliferation was determined by CFSE dilution using a FACS Canto^™^ II on day 6 after addition of activation beads.

### Flow cytometry

*In vitro* generated MDMs were detached from 6 well plates by incubation at 37°C for 30 minutes with Accutase (Pan Biotech, #P10-21100). MDMs cultured in 3D tumor cell/fibroblast co-culture spheroids were dissociated from spheroids by incubation at 37°C for 45 minutes with Accutase and carefully re-suspended by pipetting up and down every 10 minutes. A 70μm cell strainer (BD Falcon^™^, #352350) was used to remove clumps and obtain a single cell suspension. Cells were washed with PBS with 2% FCS, blocked with human IgG (Invitrogen^™^) for 15 min and stained for 30 min with conjugated antibodies or matching isotype controls. DAPI (Roche, #10236276001) staining with 200ng/ml for 10 min was performed right before measurement to discriminate dead cells. All steps were performed at 4°C (on ice). Sample acquisition was performed using a FACS Canto^™^ II (BD Biosciences) and a LSR^™^ II (BD Biosciences). To analyse the phenotype of spheroid polarized MDMs, we first gated on morphology (SSC-A vs. FCS-A) followed by gating of single viable cells (DAPI vs. FCS-W). To discriminate different cell types, EpCAM was used as tumor marker, CD11b as myeloid cell marker and cells negative for both EpCAM and CD11b expression were identified as fibroblasts. CD11b^+^ cells were then gated for further analysis of typical macrophage marker. Geometric mean fluorescence intensities (MFI) were analyzed by FlowJo 10.1 software (TreeStar Inc.).

### Immunohistochemistry

Co-culture spheroids of one 96 well plate were harvested, washed once in PBS and fixed in 4% Paraformaldehyde in PBS for 1 hour at RT. After spheroid embedding in 1% agarose in PBS and dehydration series (3x 70% ethanol, 2x 95% ethanol, 2x 100% ethanol each for 1:30 hour, 3x xylol for 1 hour, 4x paraffin for 1 hour), spheroids were embedded in paraffin and 1.5μm sections were cut using a microtome. Sections were placed on SuperFrost glass slides (ThermoFischer Scientific, #1014356190) and dried at 37°C overnight. Sections were stained by using automated staining on a BenchMark XT instrument (Ventana Medical Systems). Single marker staining was performed for CD68 (clone PG-M1, DAKO, #M0876) with chromogenic detection using diaminobenzidine (DAB) to show myeloid cell infiltration into spheroids.

### Statistics

All data are presented as mean ± SD from at least 4 independent experiments unless otherwise indicated in the figure legends. Figures were prepared using GraphPad Prism V6.04 software, Microsoft Excel 2010 and Microsoft PowerPoint 2010. Statistical significances were calculated by using an unpaired Student´s *t* test with (un)equal variances; *p < 0.05, **p < 0.01, ***p < 0.001.

## Results

### 3D co-culture of tumor cells with fibroblasts supports spheroid formation and cell survival

**Fig 1:**
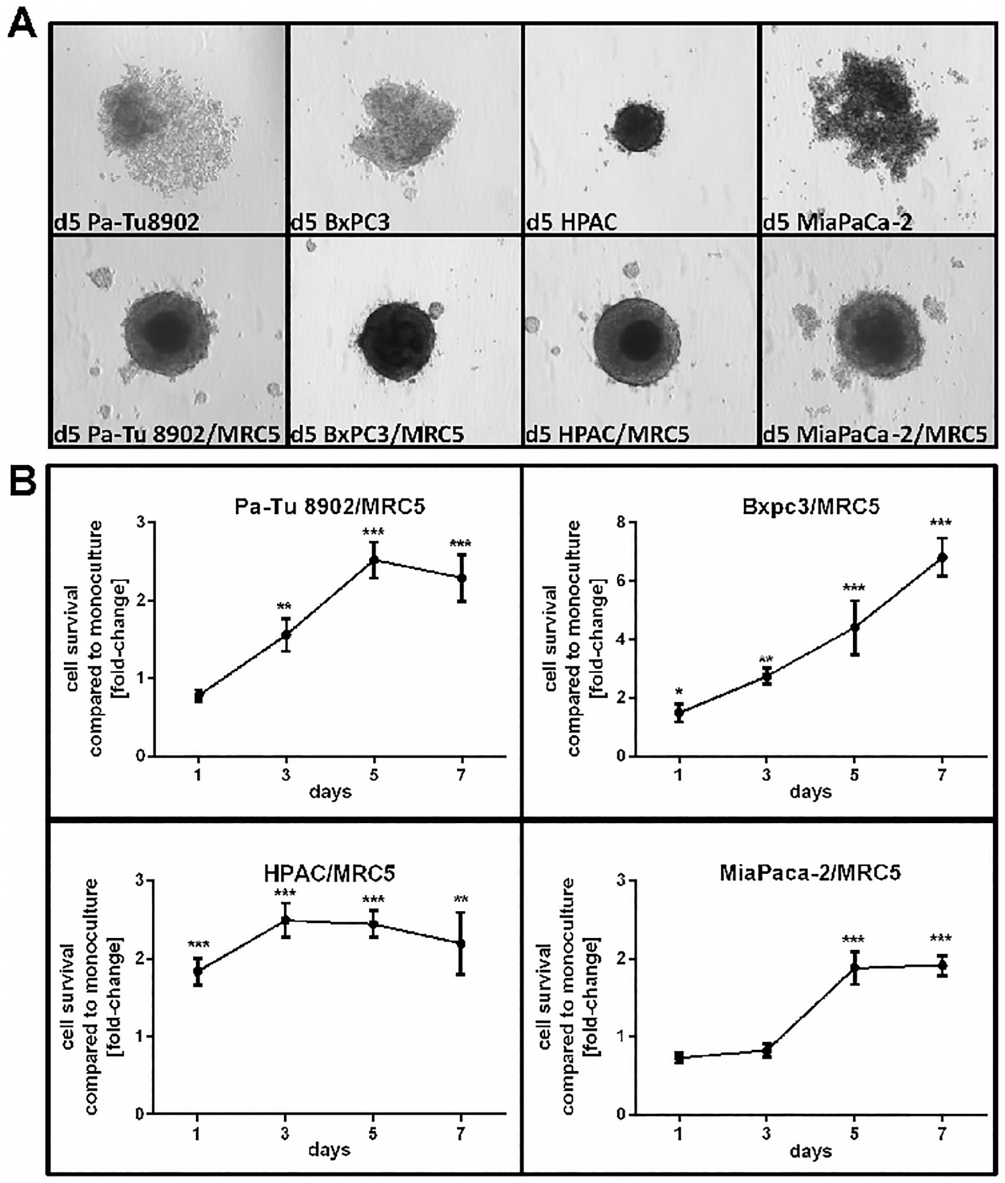
Formation and viability of tumor cell/fibroblast spheroids. Tumor cells were cultured alone or with fibroblasts in a poly-Hema-coated 96 well plate for 5 days as described before. 3D spheroid formation and cell viability was measured by CellTiterGlo assays. A) All tumor cell lines showed compact spheroid formation in co-culture with fibroblasts (MRC5) compared to tumor cell monoculture. B) Cell viability of tumor cells was strongly increased in co-culture with MRC5 and reached the maximum on day 5 for most of the cell lines. BxPC3 exhibited the greatest increase of viability upon co-culturing with MRC5, whereas the other cell lines reached a plateau at day 5. Statistical significance was calculated of n = 5 independent experiments by using an unpaired Student´s *t* test with unequal variances; *p < 0.05, **p < 0.01, ***p < 0.001.

To investigate if 3D co-culture of tumor cells with MRC5 fibroblasts affects cell viability, we co-cultured different pancreatic cancer cell lines with fibroblasts for 7 days. Co-culture of all tumor cell lines with MRC5 fibroblasts strongly enhanced the ability to form spheroids within 5 days (Fig 1A). While Pa-Tu 8902, BxPC3 and MiaPaCa-2 cultured without the MRC5 fibroblasts formed loose cell aggregates on day 5, HPAC cells formed tight spheroids in monoculture. A clearly well-defined spheroid border could be observed for all tumor cell lines in co-culture with MRC5 except for MiaPaCa-2. All tumor cell lines showed increased survival in co-culture with fibroblasts and 3 out of 4 tumor cell lines reached the highest viability on day 5 (Fig 1B) compared to tumor mono-culture. BxPC3 co-cultured with fibroblast exhibited strongly increased cell survival until day 7. These data indicate that fibroblasts support spheroid formation and cell survival.

### Monocyte addition does not influence the viability of tumor cell/fibroblast co-cultures

**Fig 2:**
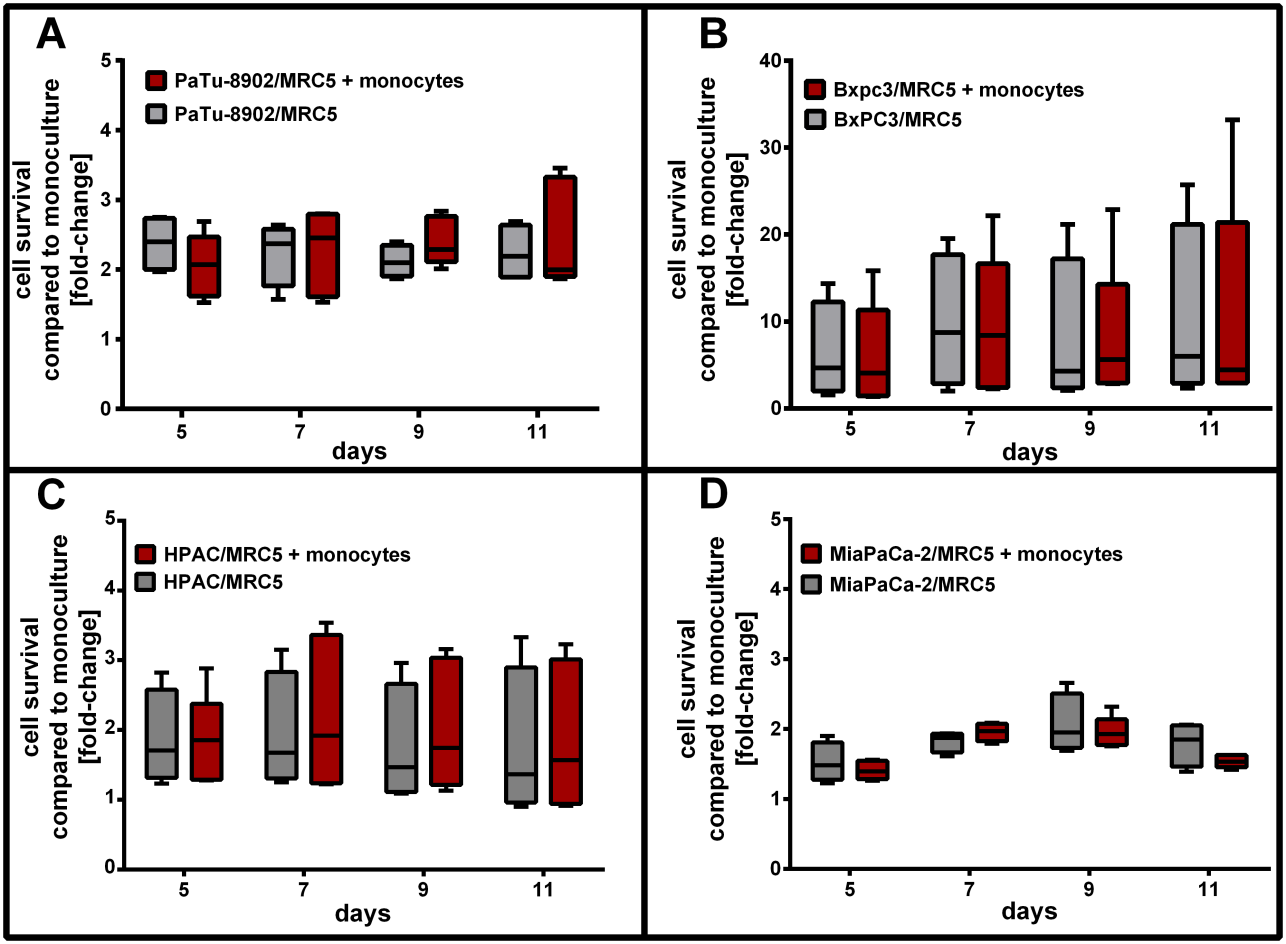
Viability of tumor cell/fibroblast co-culture with monocytes. Tumor cells were co-cultured with fibroblasts for 5 days. Freshly isolated naïve monocytes were added to tumor cell/fibroblast co-culture on day 5 and cell viability was measured every 2 days from day 5 to 11. Addition of monocytes did not influence the co-culture´s viability and tumor cell/fibroblast co-cultures with monocytes were as viable as co-cultures without monocytes for each day and tumor cell line. Represented are n = 5 independent experiments. Using an unpaired Student´s *t* test with unequal variances, no significances were observed between tumor cell/fibroblast co-cultures with and without monocytes.

**Fig 3:**
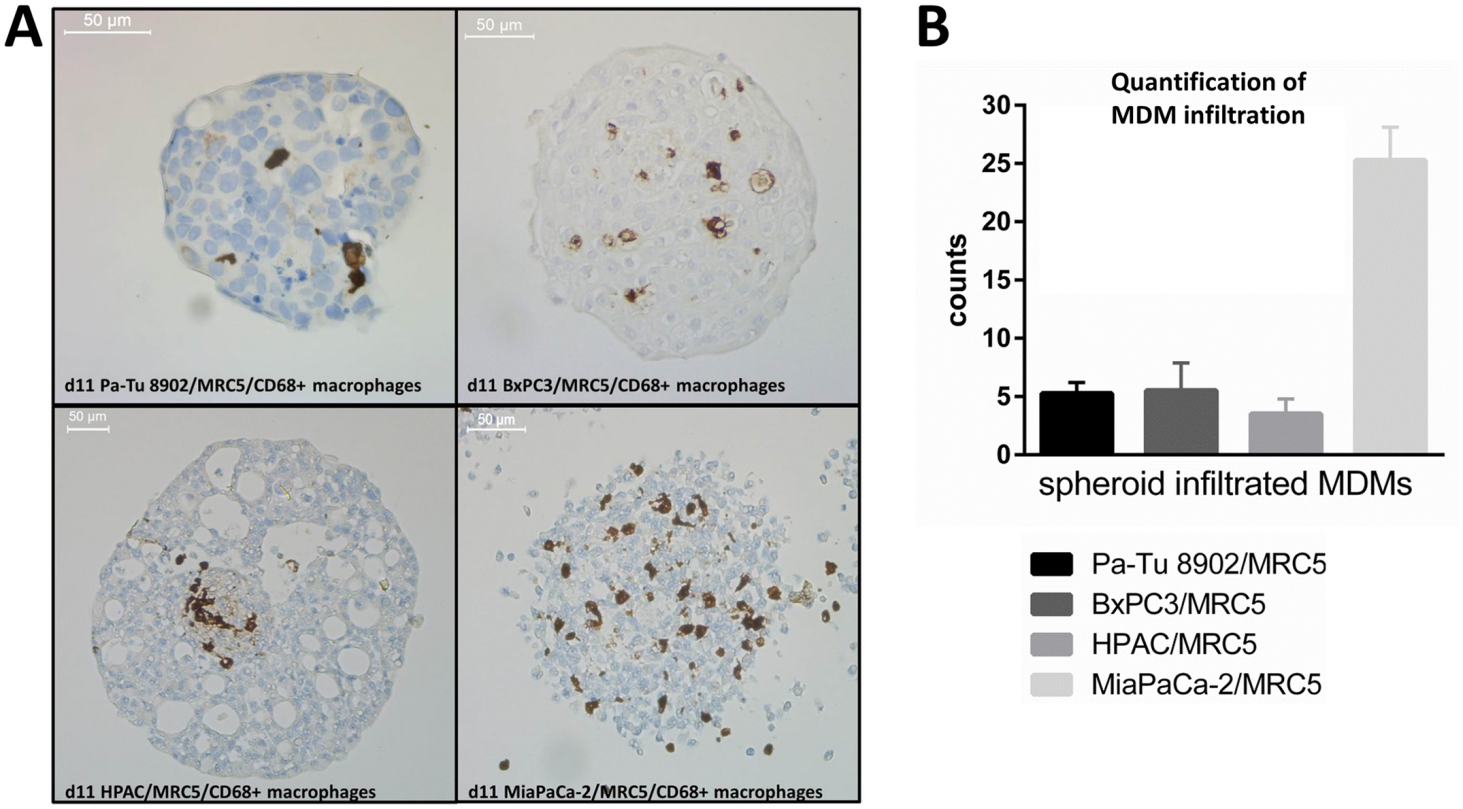
Spheroid polarized MDM infiltration into 3D co-culture spheroid. Tumor cells and fibroblasts were 3D co-cultured for 5 days to form tight spheroids. On day 5 of tumor cell/fibroblast spheroid formation, monocytes were added for 6 days to determine cell migration into spheroids. A) The pan-macrophage marker CD68 on myeloid cells was detected with DAB by performing IHC using a Benchmark XT instrument. Infiltration of spheroid polarized MDMs can be observed for all co-cultures, but was highest in MiaPaCa-2/MRC5 co-culture. One representative picture is shown for each tumor cell line. B) Quantitative analysis of spheroid infiltrated MDMs of n = 4 independent experiments.

To determine the influence of monocyte addition to co-culture viability and proliferation, tumor cells and fibroblasts were co-cultured for 5 days to form spheroids and freshly isolated monocytes were added (S1 Fig). Tumor cell/fibroblast/monocyte co-cultures were incubated for 6 days. The viability of the co-culture spheroids with monocytes was measured every 2 days starting from day 5 by using CellTiterGlo (Fig 2). Although monocytes infiltrated all the tumor cell/fibroblast spheroids (Fig 3), viability of co-cultures was comparable to co-cultures without monocytes. While monocytes alone did not survive in the culture medium in poly-Hema coated wells (S2 Fig), they were still viable in co-cultures and did not positively influence the viability of co-cultures (Fig 2). However, there was no reduction in viability of tumor cell/fibroblast co-cultures observed after addition of monocytes and the co-culture remained stable and viable throughout 11 days.

### Tumor cell/fibroblast spheroids promote M2-like polarization of monocytes in 3D co-culture

**Fig 4:**
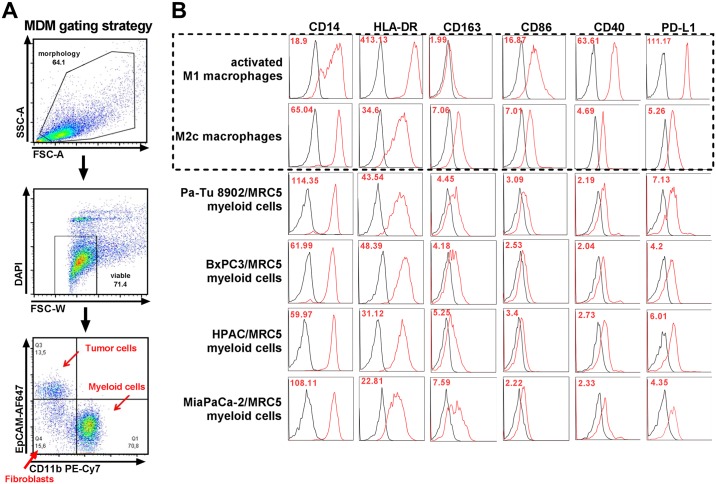
3D co-culture polarized MDMs resemble M2-like macrophages. Tumor cells and fibroblasts were co-cultured for 5 days. Monocytes were added to co-culture on day 5 to differentiate for 6 days. Spheroids were collected and dissociated by using Accutase to obtain a single cell suspension. A) Single cell suspensions were analysed by flow cytometry using the illustrated gating strategy for MDM phenotyping. B) Cell surface marker expression of 3D myeloid cells was compared to *in vitro* generated M2c and activated M1 macrophages. Typical M2 and M1 macrophage marker were analyzed by flow cytometry. 3D co-culture MDMs expressed high levels of CD163 and CD14 and low levels of CD86 and HLA-DR comparable to *in vitro* differentiated M2 macrophages (dotted box). Shown is one representative out of n = 5 experiments, red numbers show geometrical mean values for relevant markers.

Myeloid cells infiltrating the spheroids were identified by immunohistochemistry staining with CD68, a pan macrophage marker. CD68^+^ myeloid cell infiltration into spheroids was observed in all co-culture spheroids, although at different rates (Fig 3). MiaPaCa-2/MRC5 formed compact spheroids but did not have a clear border compared to the other tumor cell lines. These spheroids were strongly infiltrated by CD68^+^ myeloid cells compared to other co-culture spheroids (Fig 3B). These data suggested that tumor cell/fibroblast spheroid tightness may be a factor that influences the migration of monocytes into the spheroid. The phenotype of viable monocytes that infiltrated the tumor cell/fibroblast spheroids was then characterized by analysing the expression of cell surface markers by flow cytometry (Fig 4). Typical M2 (CD14, CD163, Arginase-1) and M1 (CD86, HLA-DR, CD40) macrophage marker were measured on the spheroid polarized MDMs and compared to *in vitro* generated M2c macrophages and activated M1 macrophages (S1 Table). As expected, M1 macrophages expressed low levels of CD163 and CD14. In addition, they expressed higher levels of activation marker CD86, HLA-DR, CD40 and the checkpoint molecule PDL1. M2 macrophages, on the other hand, expressed high levels of CD163 and CD14, lower levels of CD86, HLA-DR, CD40 and PDL1. Arginase-1, an M2 macrophage marker which is involved in inhibition of nitric oxide (NO) production, was expressed only by the *in vitro* generated M2 macrophages. The spheroid polarized MDMs expressed low levels of CD86, HLA-DR and CD40 and high levels of CD14, CD163 and Arginase-1. The expression pattern was similar to the *in vitro* generated M2 macrophages.

### Spheroid polarized MDMs induce differential cytokine secretion in a cell line dependent manner

**Fig 5:**
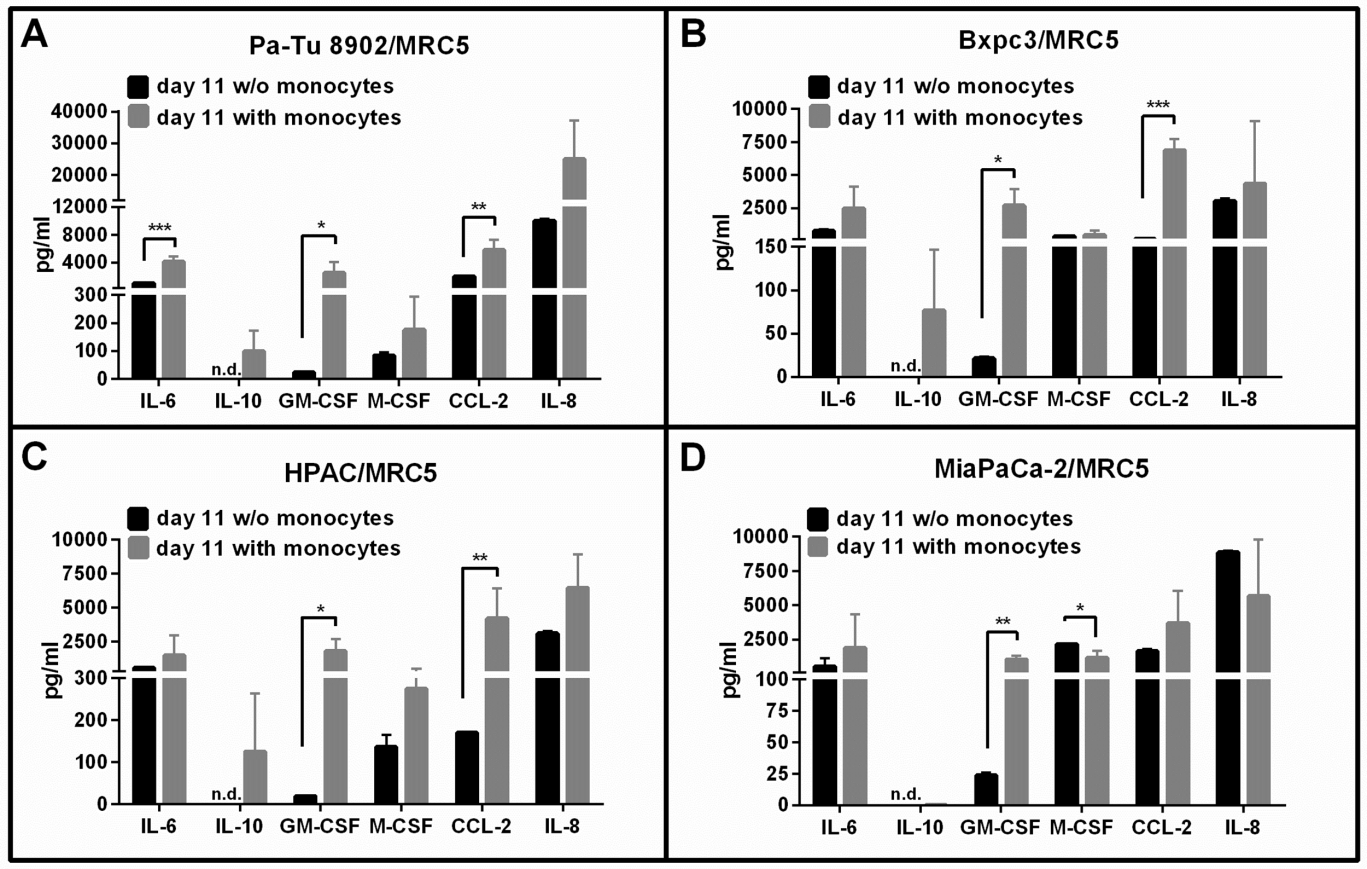
3D tumor cell/fibroblast co-culture with monocytes induces differential secretion of cytokines, chemokines and growth factors. Tumor cells and fibroblasts were co-cultured for 5 days. Monocytes were added to co-culture on day 5 and further cultivated for 6 days. Supernatants were collected on day 11 from co-cultures without and with monocytes. A panel of 19 soluble factors was measured using Luminex multiplex technology and the most relevant ones at detectable levels are shown. Increased levels of several cytokines and chemokines were detected on day 11 after addition of monocytes. Statistical significance was calculated of n = 3 independent experiments by using an unpaired Student´s *t* test with unequal variances; *p < 0.05, **p < 0.01, ***p < 0.001.

Soluble factors including cytokines, chemokines and growth factors in supernatants from tumor cell/fibroblast co-culture was measured prior to and 6 days after monocyte addition by using the Luminex multiplex technology. We observed that tumor cell/fibroblast spheroids differed in their cytokine/growth factor profile depending on the tumor cell line (Fig 5). However, all tumor cell lines co-cultured with MRC5 fibroblasts secreted high levels IL-6, CCL-2 and IL-8 and low levels of IL-10. We further observed that the levels of IL-6, CCL-2 and GM-CSF increased significantly in most co-culture supernatants on day 6 after monocyte addition. IL-10 was not detected on day 11 of tumor cell/fibroblast co-culture, but was induced when monocytes were added to the co-culture. While BxPC3/MRC5 and MiaPaCa-2/MRC5 co-culture with monocytes showed strongly increased levels of M-CSF comparable to co-culture without monocytes, the addition of monocytes to Pa-Tu 8902/MRC5 and HPAC/MRC5 co-cultures induced increased secretion of M-CSF compared to co-culture without monocytes. Pro-inflammatory cytokines such as IFN-γ, IFN-α and IL-2 as well as tumor-promoting factors like TGF-α were not detectable in our co-culture model (S2 Table). These data showed that apart from the tumor cell line used, the addition of monocytes to the co-cultures influenced the cytokine/growth factor profile of the 3D co-cultures.

### Spheroid polarized MDMs inhibit T cell proliferation in co-culture

**Fig 6:**
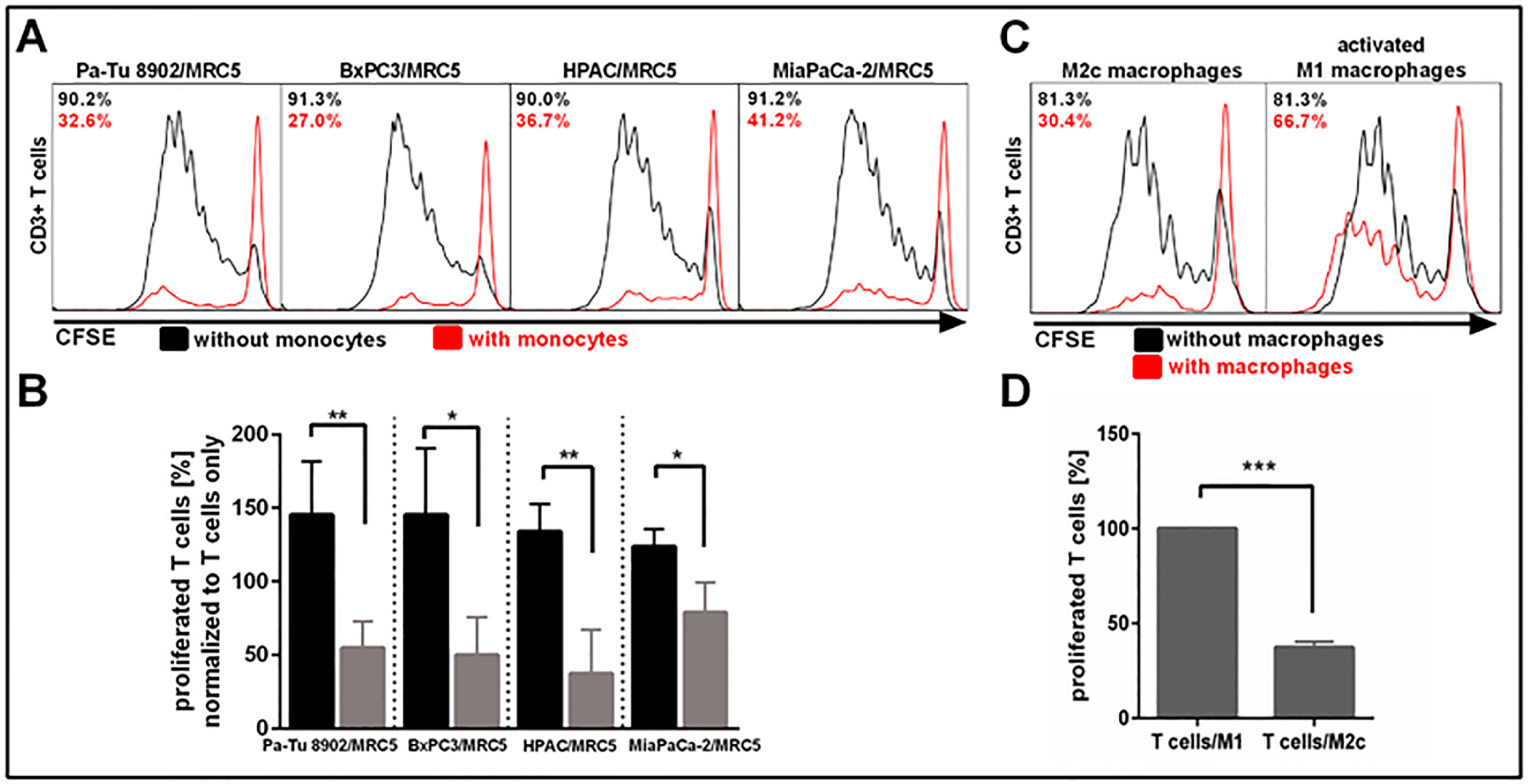
3D co-culture polarized MDMs suppress CD3+ T cell proliferation. Tumor cells and fibroblasts were co-cultured for 5 days to form tight spheroids. Freshly isolated monocytes were added on day 5 to differentiate for 6 days. Autologous CD3+ T cells were labeled with CFSE on day 11, added to co-cultures with and without monocytes and stimulated with CD3/CD28 activation beads. Proliferation was measured after 6 days using flow cytometry. A) T cells proliferated strongly in co-culture with tumor cell and fibroblasts, but were suppressed in co-culture with spheroid polarized MDMs. B) Strongest suppression of T cell proliferation was observed in Pa-Tu 8902/MRC5 and HPAC/MRC5 co-cultures with spheroid polarized MDMs. C, D) CD3+ T cell proliferation was more effectively suppressed in co-culture with *in vitro* generated M2c macrophages compared to co-culture with activated M1 macrophages. Statistical significance was calculated of n = 4 independent experiments by using an unpaired Student´s *t* test with unequal variances; *p < 0.05, **p < 0.01, ***p < 0.001.

We found that spheroid polarized MDMs expressed M2-like macrophage cell surface markers and also secreted factors that have an immunosuppressive function, which led us to investigate if these cells had the functional ability to inhibit T cell activation and proliferation. Data from CFSE based CD3/CD28 bead activated T cell proliferation assays showed that T cell proliferation was indeed strongly inhibited (27%–41% of proliferating T cells) in co-cultures containing spheroid polarized MDMs (Fig 6A). T cell suppression capability of the spheroid polarized MDMs was comparable to the *in vitro* generated M2c macrophages (30% proliferating T cells) in contrast to activated M1 macrophages (66% proliferating T cells) (Fig 6C). Only 40% T cells proliferated in co-culture with M2c macrophages compared to M1 macrophages (Fig 6D). These findings indicate that spheroid polarized MDMs co-cultured with pancreatic tumor cell lines and MRC5 fibroblasts not only showed an M2-like macrophage phenotype in terms of cell surface marker expression and cytokine profile, but also functionally suppress T cell proliferation.

### Activation of CD4^+^ and CD8^+^ T cells is impaired in the presence of spheroid polarized MDMs in 3D co-culture

**Fig 7:**
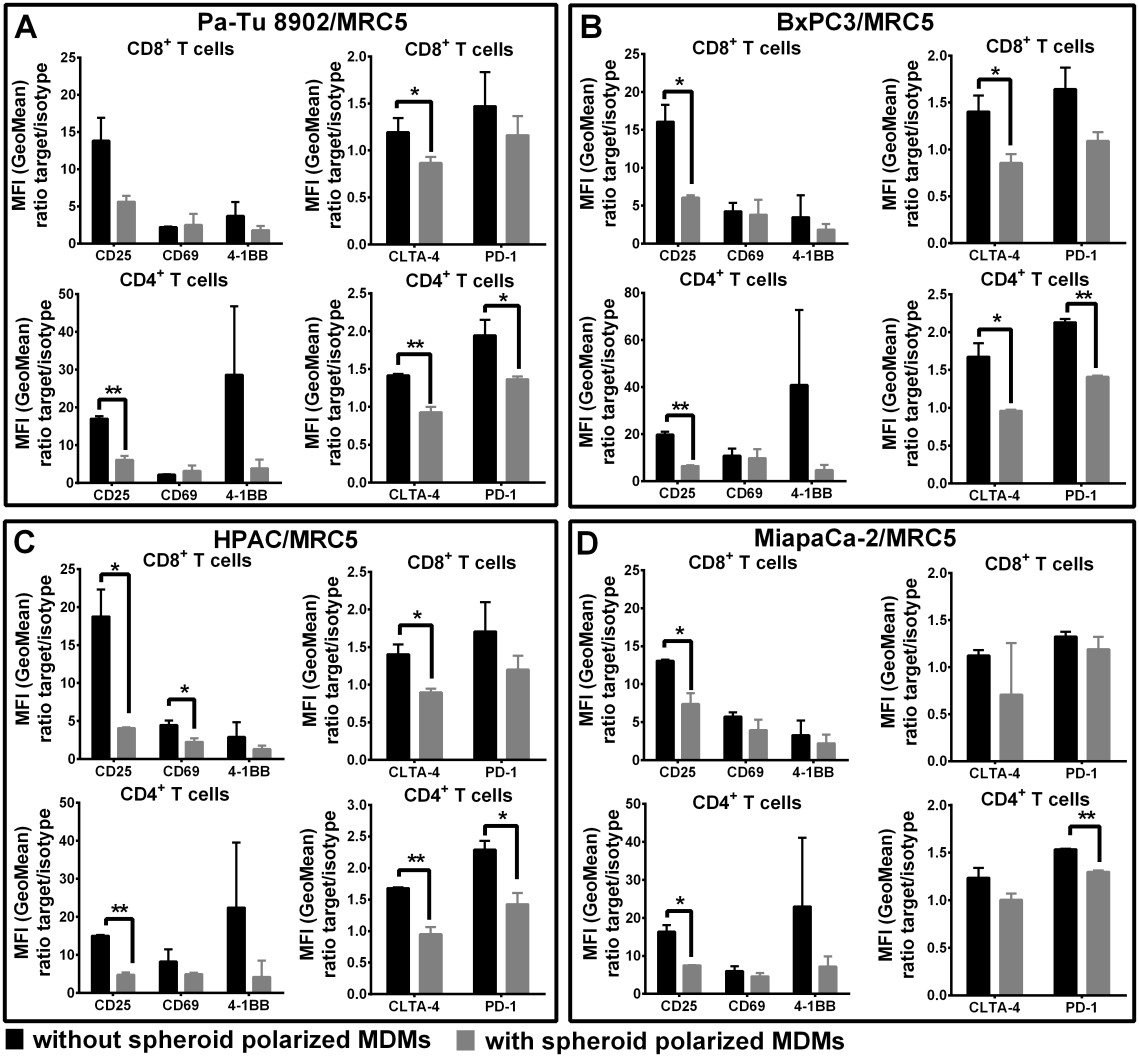
Expression of cell surface activation and checkpoint marker on CD8+ and CD4+ T cells decreases in co-culture with 3D co-culture polarized MDMs. Tumor cells and fibroblasts were co-cultured for 5 days to form tight spheroids. Freshly isolated monocytes were added on day 5 to differentiate for 6 days. Autologous CD8+ and CD4+ T cells were added to co-cultures with and without spheroid polarized MDMs on day 11 and stimulated with CD3/CD28 activation beads. Cell surface marker expression of CD8+ and CD4+ T cells was measured 6 days after T cell activation by flow cytometry. Statistical significance was calculated of n = 3 independent experiments by using an unpaired Student´s *t* test with unequal variances; *p < 0.05, **p < 0.01, ***p < 0.001.

After observing a strong inhibition of proliferation in T cells, we investigated the phenotypic changes that occurred in the T cells upon co-culture at different time points of CD3/CD28 mediated activation. We evaluated expression of early activation markers CD25 and CD69, immune-modulatory molecules like 4-1BB and checkpoint molecules PD1 and CTLA4. Early activation markers, CD69 and CD25 were both upregulated upon T cell stimulation in co-culture with tumor cell/fibroblasts. In the presence of spheroid polarized MDMs however, the expression for CD25 was significantly lower on both CD4^+^ and CD8^+^ T cell sub-populations (Fig 7). A clear but non-significant decrease in expression of CD69 was also observed in both CD4^+^ and CD8^+^ T cell. Further, we observed a decrease in the expression of the activating co-stimulatory molecule 4-1BB up to 5-fold on CD4^+^ T cells and 2-fold on CD8^+^ T cells in the presence of spheroid polarized MDMs in comparison to activated T cells in co-culture with tumor cells and fibroblasts alone. Surface expression of the immune check point molecules PD-1 and CLTA-4 was significantly down-regulated in the presence of spheroid polarized MDMs (Fig 7). Taken together, we observed that spheroid polarized MDMs functionally inhibited CD4^+^ and CD8^+^ T cells reflected by decreased expression of activation markers.

### Treatment of spheroid polarized MDMs with immune modulating compounds partially restores T cell proliferation in 3D co-culture

**Fig 8:**
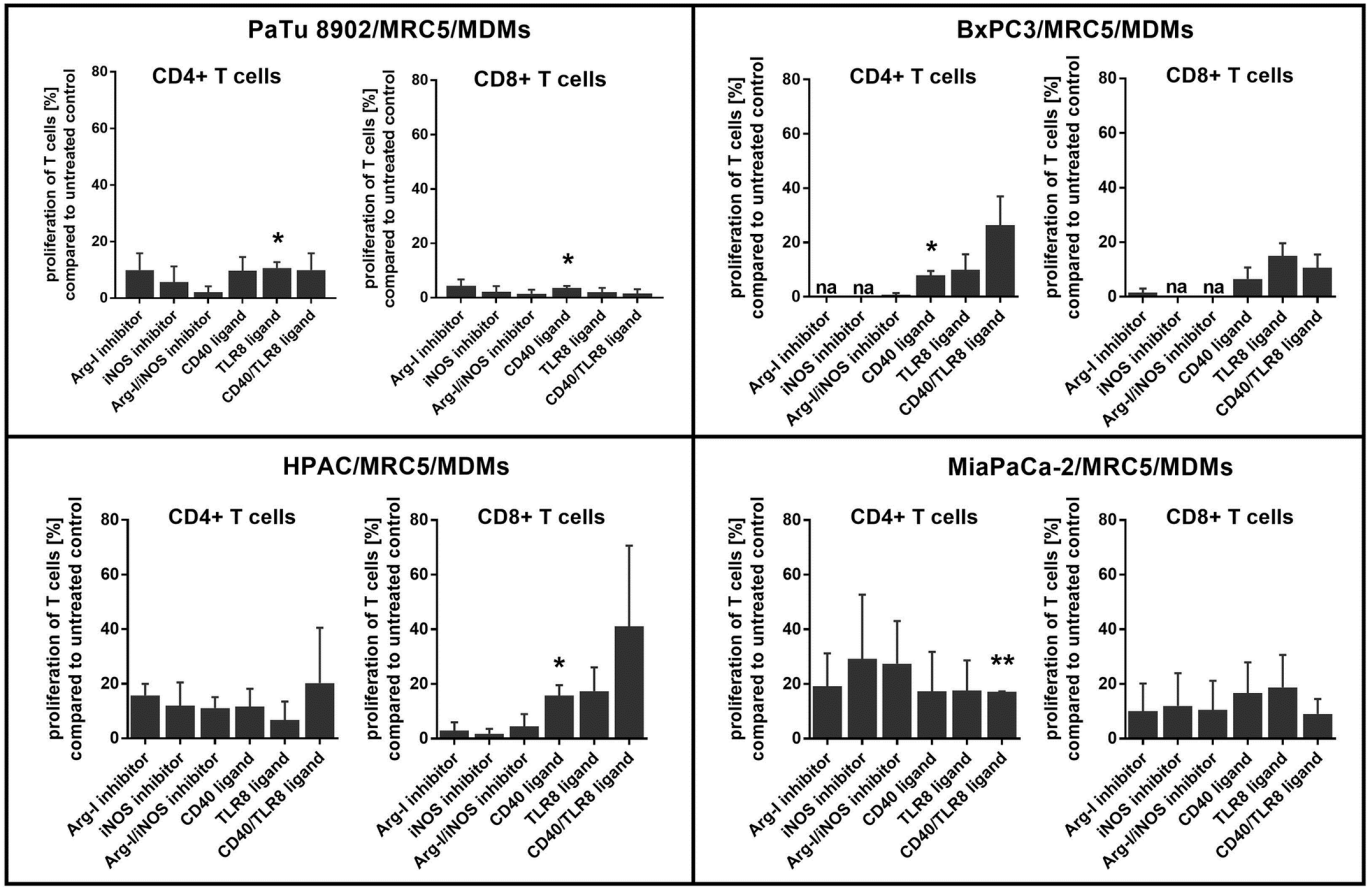
Treatment of spheroid polarized MDMs with immune modulating compounds partially restores T cell proliferation in 3D co-culture. Tumor cells and fibroblasts were co-cultured for 5 days to form tight spheroids. Freshly isolated monocytes were added on day 5 to differentiate for 6 days. One day prior T cell addition, co-cultures were treated with inhibiting or activating compounds either alone or in combination and compared to untreated T cells. Autologous CD3+ T cells were labeled with CFSE on day 11, added to co-cultures with and without spheroid polarized MDMs and stimulated with CD3/CD28 activation beads. Proliferation was measured after 6 days using flow cytometry. Statistical significance was calculated of n = 3 independent experiments by using an unpaired Student´s *t* test with unequal variances; *p < 0.05, **p < 0.01, ***p < 0.001.

As we observed suppressed T cell proliferation induced by spheroid polarized MDMs, we evaluated if we could revert T cell suppression by using CD40 and TLR8 ligands to re-activate spheroid polarized MDMs as well as Arginase-I and iNOS inhibitors to block T cell suppressing enzymes. For all compounds used, we could show a tendency of cell line dependent increased CD4^+^ and CD8^+^ T cell proliferation compared to T cells in untreated 3D co-culture, which however is non-significant (Fig 8). Significant changes could only be observed for the activating molecules CD40 ligand and TLR8 ligand. The inhibiting molecules Arginase-I and iNOS only showed a slight, but non-significant cell-line dependent reversion of T cell proliferation for both CD8^+^ and CD4^+^ T cells co-cultured with tumor cells, fibroblasts and spheroid polarized MDMs. T cells co-cultured with BxPC3/MRC5/MDMs did not show any proliferation after treatment with inhibiting molecules compared to untreated T cells. The combination of compounds did not lead to improved T cell proliferation in comparison to single compound treatment. Although no statistically significant differences could be detected regarding treatment with inhibitors, we observed a donor dependent variation in reversal of T cell proliferation. The activating molecules, CD40 and TLR8 ligand, seemed to enhance T cell proliferation for both CD8^+^ and CD4^+^ T cells co-cultured with tumor cells, fibroblasts and spheroid polarized MDMs stronger than the inhibiting molecules (Fig 8). For T cells co-cultured with HPAC/MRC5 and MDMs, the combination of CD40/TLR8 ligands did seem to be slightly more effective than the single agent treatment.

## Discussion

Immune cell infiltrates in primary tumors have been shown to correlate with tumor progression. Therapies targeting immune cells in cancer are now in the clinic and have shown unprecedented results in terms of responses and overall survival [15]. However, a vast majority of patients still remain non-responsive or are resistant to these therapies [16]. It is therefore necessary to understand the mechanisms operating in this context to define suitable therapies and overcome resistance. Crosstalk between different cells in the tumor microenvironment plays an important role in tumor growth and tumor-mediated immune suppression. Finding a suitable therapeutic setting that helps patients with pancreatic cancer has been particularly difficult given the unique characteristics of the tumor and its immune composition. Pre-clinical studies to test different therapeutic strategies include both *in vivo* mouse models and *in vitro* studies. Both methods have their own advantages and drawbacks. Although mouse models are closer to patient tumors, differences between mouse and human immune mechanisms and lack of cross reactivity limit the relevance of these experiments. Reflecting the *in vivo* situation in a superior way compared to 2D monolayers, 3D co-culture systems are able to provide critical insight into the role of the inflammatory tumor microenvironment and its interaction between desmoplastic fibroblasts as well as with immune cells in cancer progression and therapy resistance. Studies have already reported that drug responsiveness is limited in 3D systems compared to 2D monolayers [17, 18]. To establish 3D models, most studies focus on one or two cell lines to observe cell-cell interaction, but to achieve a more realistic *in vivo* situation, not only tumor cells and fibroblasts, but also immune cells have to be kept into account [19]. Fibroblasts and immune cells have been reported to play a key role in tumor initiation, progression and metastasis of PDAC and various strategies including immunotherapies are currently being tested [20–22]. Tumor-associated macrophages, in particular, have been linked to poor prognosis in more than 80% of analysed cancer types [23, 24]. We previously established a 3D co-culture model to investigate crosstalk between pancreatic cancer cells and fibroblasts and showed that the model reflected clinical situation and influenced therapeutic response *in vitro* [13]. In this study, we extended this model by adding monocytes to examine the influence of the crosstalk between tumor cells, fibroblasts and monocytes on monocyte differentiation and function (S1 Fig). So far, studies have shown that TAMs support tumor growth rather indirectly by generating an immunosuppressive milieu and producing pro-tumorigenic cytokines such as the epidermal growth factor (EGF) [25, 26]. Upon addition of monocytes to our 3D tumor cell/fibroblast co-culture, we could not observe a direct effect of spheroid polarized MDMs on tumor growth and survival, which led us to systematically analyse the cytokine profile of the co-cultures, phenotype of the monocytes and their functional influence on T cells. GM-CSF and M-CSF along with other cytokines like IL-6 and IL-8 have been shown to be involved in recruitment and differentiation of myeloid cells into M2 macrophages and myeloid derived suppressor cells (MDSCs) in the tumor apart from promoting tumor angiogenesis [25]. IL-10 is well known for its role in M2 macrophage differentiation and suppression of various myeloid cells and T cells. These cytokines were detected in the supernatants of our co-culture model and have been shown to be present in PDAC patients [27, 28]. The presence of these cytokines has been associated with poor performance status and worse prognosis [29, 30]. Not only has the presence of specific soluble molecules been associated with worse clinical outcomes, but also immune cell infiltration into the tumors. High densities of TAMs have been linked to increased angiogenesis, differentiation of cancer cells and elevated levels of tumor-promoting cytokines [31]. In our model, we could show the infiltration of spheroid polarized MDMs in a cell line dependent manner, being another step closer to developing a more realistic 3D *in vitro* tumor model. Furthermore, previous studies reported that the phenotypes of TAMs often differ based on the location and the stage of the tumor [32]. TAMs in PDAC are defined as CD68^+^ CD163^+^ and CD2014^+^ positive and are associated with lymphatic metastasis [4]. High numbers of infiltrated TAMs have been shown to correlate with poor prognosis [4, 33]. It has also been shown that TAMs show high expression of CD14 and to some extent expression of HLA-DR and CD86 in breast cancer [34]. Expression of type-I Arginase, which is known to promote tumor growth by suppression of effector T cells, has been described as a marker of M2-like macrophages [35, 36]. We observed in our 3D co-culture model that tumor/fibroblast spheroids induced an M2 polarization of co-cultured monocytes with a CD14^+^ CD163^+^ HLA-DR^low^ CD86^low^ ARG-1^+^ phenotype which resembles the phenotype of TAMs in PDAC. It has been reported that tumor infiltrating lymphocytes (TILs) are functionally defective or incompletely activated since often the presence of TILs, mostly T cells, does not prevent tumor growth and progression [37]. Analysing the activation status of T cells, it has been shown that high expression of the T cell activation marker CD25 (Interleukin 2 receptor α) on T cells in cutaneous malignant melanoma correlates with longer survival. Low expression of CD25 however, indicated functional impairment of T cells, whereas the early T cell activation marker, CD69, could not be used to successfully predict the T cell activation status [38, 39]. These data are in line with our findings that the activation of T cells in co-culture with the spheroid polarized MDMs in our model down-regulate CD25 and CD69 indicating impaired activation of these cells. Other immune check point/modulatory markers like 4-1BB; PD1 and CTLA4 are known to be upregulated upon T cell activation. 4-1BB is reported to be a biomarker for tumor-reactive T cells which are able to inhibit tumor growth *in vivo* [40]. T cells expression of PD-1 and CTLA-4 showed impaired T cell function during the effector phase when engaged with their ligands and restrain anti-tumor immunity [41]. These markers were down-regulated in our co-culture model when T cells were co-cultured with spheroid polarized MDMs but not when co-cultured with 3D tumor cell/fibroblast spheroids only. TAMs have also been reported to not only suppress T cell activation but also inhibit proliferation of T cells in the tumor microenvironment *in vivo* [42, 43]. Presence of spheroid polarized MDMs in our co-culture model not only suppressed T cell activation as reflected by low expression of T cell activation markers, but also inhibited T cell proliferation in our *in vitro* system. These data show that our co-culture model created a suitable niche to mimic *in vivo* conditions and induced *in vivo* like phenotype in the cell types involved.

Due to limited success in treatment of PDAC cancer with standard of care therapy like chemo-, radiation therapy as well as surgery, cancer immunotherapy has been the method of choice in the recent past. To overcome immune suppression, studies focused on targeting checkpoint molecules such as PD-1 and CTLA-4. As mentioned earlier, we observed a down-regulation of checkpoint molecules like PD1 and 4-1BB on MDM co-cultured T in our model. This may be important information regarding the phenotype of the infiltrating T cells in PDAC. If this is validated by examining patient samples it may suggest that treatment with anti-PD1 or 4-1-BB therapies may not be efficacious in these tumors. On the other hand pro-inflammatory receptors such as CD40 to reverse immunosuppressive phenotype of M2-like macrophages into pro-tumoral M1 macrophages are being performed [15]. Anti-CD40 antibody, for example, has been shown to re-activate macrophages in pancreatic cancer mouse models, leading to the recruitment of anti-tumor M1 macrophages to the tumor site, depletion of tumor stroma and consequently to increased efficacy of chemotherapeutic agents such as gemcitabine [44]. In our 3D co-culture model, we observed increased, T cell proliferation by CD40 ligand and with Toll-like receptor (TLR) 8 ligand treatment. We also observed a similar trend in samples treated with the Arginase-1 and the iNOS inhibitors. These effects were cell line specific and also did not reach statistical significance due to high donor variation. It would be necessary to increase the number of donors to get a clearer picture.

To date, clinical trials with cancer immunotherapy molecules such as anti-PD-1 and anti-CTLA-4 ended with disappointing outcomes, emphasizing the need for combination therapies that focus on multiple immune cells and checkpoint markers to treat tumors of patients who do not respond to single agent therapies [15, 16]. However, the expression level of these molecules is rather low on T cells and myeloid cells respectively in our co-culture model. Based on these data one could speculate that targeting PD1/PDL1 or CTLA4 would not be a successful approach. Instead, a combination therapy starting with molecules that increase the expression of the above mentioned molecules (for e.g. CD40 activation) or remove the suppressive factors/cells (IL-10 inhibition or depletion of M2 macrophages by targeted therapy like CSF1R) would be more efficacious. The presence of different cell types in our model provides the opportunity to test other interesting immune modulating agents in this system to study their impact on the monocyte and T cell phenotype in this system.

Our 3D co-culture model is a promising pre-clinical tool to study the influence of immunotherapies on the different cell types, but comes along with potential limitations. Although it includes different cell types, it is still an *in vitro* model and cannot reflect the complexity of the *in vivo* tumor microenvironment. In the context of PDAC, many other cell types and factors are involved in the process leading to immune escape. For example; granulocytes or granulocytic MDSCs which have been reported to be present in the PDAC microenvironment are absent in our system. 3D models including ours are still simplistic and are not able to represent all ECM components as well as angiogenic factors [45]. The establishment of stable *in vitro* systems is difficult when using primary cells, but can also be a chance to better investigate the cause of why patients respond differently to certain types of treatment. Advances in this field of research have led to the development of bio-engineered multi-organ tissues that may provide a more realistic and predictive approach to assess drug toxicity and investigate cell-cell interactions more closely [46]. *Ex vivo* culture of tumor fragments in bioreactors or suitable culture systems are also being developed and used. These systems are very close to the patient situation and also offer flexibility in analysing various cell types. 3D co-culture systems, whether cell based systems like our model or *ex vivo* fragment based models, are a reliable tool to study the complex crosstalk between the different cell types in the tumor. Future studies focused on understanding resistance mechanisms to existing immunotherapies and identification of alternative mechanisms for therapeutic targeting will indeed be very useful to develop new therapeutic strategies in the war against cancer.

## Supporting information

S1 Fig Experimental set up of 3D co-culture model. 2000 tumor cells and 3000 MRC5 fibroblasts per well were seeded in a round-bottom poly-Hema coated 96 well plate in a total volume of 100μl. Plates were centrifuged at 300xg for 4 min and carefully transferred to the incubator at 37°C and 5% CO_2_. After 5 days, spheroids formed and 10.000 monocytes freshly isolated from healthy blood donors were added for further 6 days. On day 11, spheroids were collected for appropriate analysis.
(TIF)

S2 Fig Viability and spheroid formation of MRC5 fibroblasts and monocytes alone. 10.000 freshly isolated monocytes and 5000 MRC5 fibroblasts were seeded and cultivated in a poly-Hema coated 96 well round-bottom plates for 11 days. Cell viability was measured every 2 days from day 1 to 11 using CellTiterGlo Luminescence. MRC5 fibroblasts formed tight spheroids by day 5, but viability of the monoculture strongly decreased during this time. Monocytes formed loose cell aggregations. Monocyte viability also decreased rapidly until day 11. Represented is the mean of n = 5 independent experiments.
(TIF)

S1 Table Mean fluorescence intensities (MFI) of M1/M2 macrophage marker. Tumor cells and fibroblasts were co-cultured for 5 days. Monocytes were added to co-culture on day 5 to differentiate for 6 days. Spheroids were collected and dissociated by using Accutase to obtain a single cell suspension. Cell surface marker expression of 3D myeloid cells was compared to *in vitro* generated M2c and activated M1 macrophages. Typical M2 and M1 macrophage marker were analyzed by flow cytometry. 3D co-culture myeloid cells expressed high levels of CD163, CD14 and Arg-1 and low levels of CD86, CD80 and HLA-DR comparable to *in vitro* differentiated M2c macrophages. Represented is the geometrical mean for each target (target/isotype) with n = 5 experiments.
(TIF)

S2 Table Summary of differentially expressed cytokines and chemokines in the supernatant of 3D tumor cell/fibroblast co-cultures with and without spheroid polarized MDMs. Tumor cells and fibroblasts were co-cultured for 5 days. Monocytes were added to co-culture on day 5 and further cultivated for 6 days. Supernatants were collected on day 5 before monocyte addition and on day 11 from co-cultures without and with monocytes. A panel of 19 soluble factors was measured using Luminex multiplex technology or ELISA. Increased levels of several cytokines and chemokines could be detected on day 11 after addition of monocytes (n.d. = not detectable). Shown is the mean concentration in pg/ml of n = 3 independent experiments.
(TIF)
