# Supplementary figures and images for: Pancreatic cancer cell/fibroblast co-culture induces M2 like macrophages that influence therapeutic response in a 3D model
Source: PLoS One. 2017 Jul 27;12(7):e0182039. doi: 10.1371/journal.pone.0182039 (PMC5531481; doi:10.1371/journal.pone.0182039)

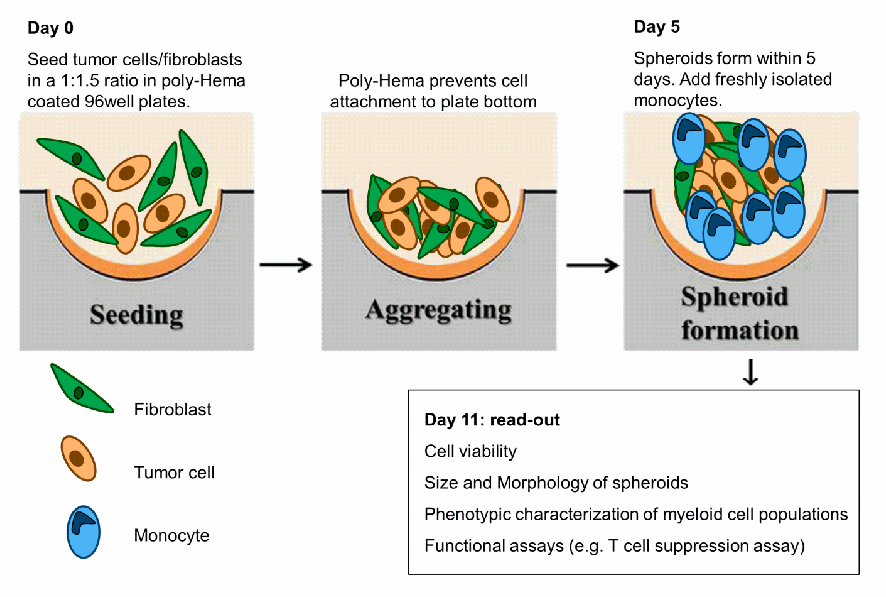

Supplement: S1 Fig — 2000 tumor cells and 3000 MRC5 fibroblasts per well were seeded in a round-bottom poly-Hema coated 96 well plate in a total volume of 100μl. Plates were centrifuged at 300xg for 4 min and carefully transferred to the incubator at 37°C and 5% CO2. After 5 days, spheroids formed and 10.000 monocytes freshly isolated from healthy blood donors were added for further 6 days. On day 11, spheroids were collected for appropriate analysis. (TIF) [file pone.0182039.s001.tif]

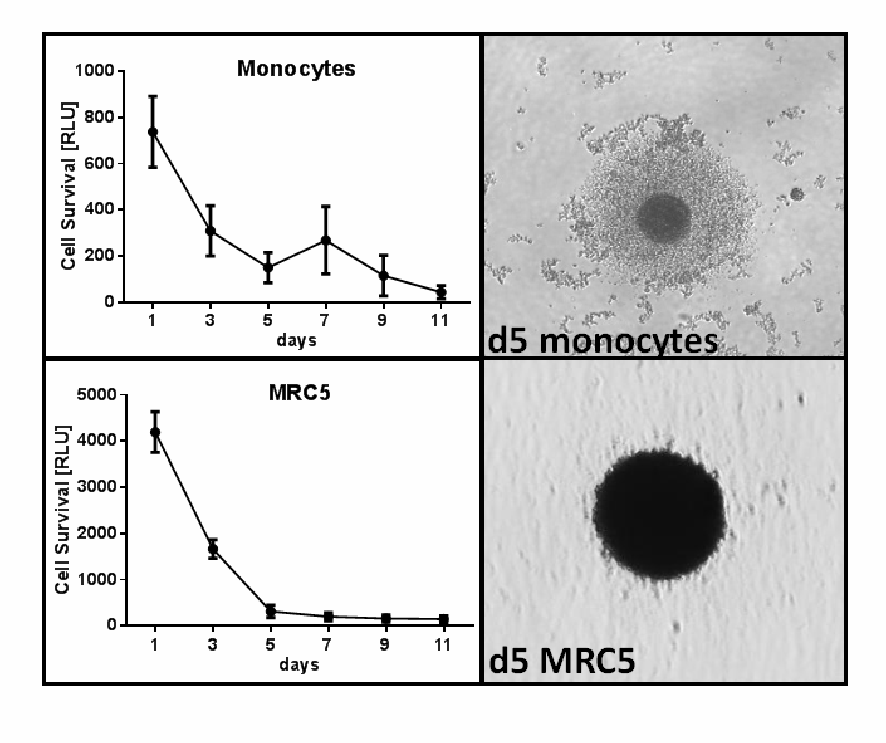

Supplement: S2 Fig — 10.000 freshly isolated monocytes and 5000 MRC5 fibroblasts were seeded and cultivated in a poly-Hema coated 96 well round-bottom plates for 11 days. Cell viability was measured every 2 days from day 1 to 11 using CellTiterGlo Luminescence. MRC5 fibroblasts formed tight spheroids by day 5, but viability of the monoculture strongly decreased during this time. Monocytes formed loose cell aggregations. Monocyte viability also decreased rapidly until day 11. Represented is the mean of n = 5 independent experiments. (TIF) [file pone.0182039.s002.tif]

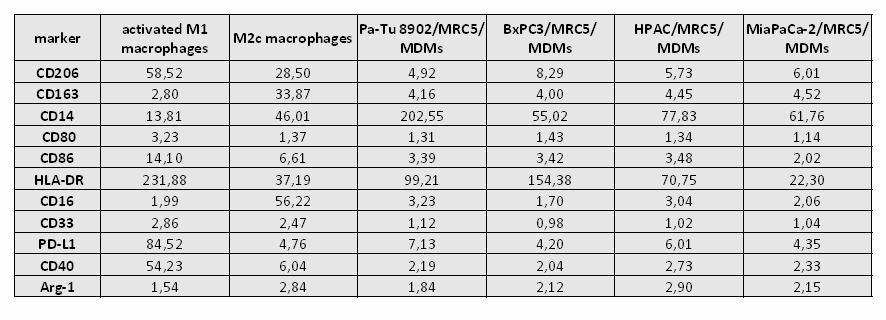

Supplement: S1 Table — Tumor cells and fibroblasts were co-cultured for 5 days. Monocytes were added to co-culture on day 5 to differentiate for 6 days. Spheroids were collected and dissociated by using Accutase to obtain a single cell suspension. Cell surface marker expression of 3D myeloid cells was compared to in vitro generated M2c and activated M1 macrophages. Typical M2 and M1 macrophage marker were analyzed by flow cytometry. 3D co-culture myeloid cells expressed high levels of CD163, CD14 and Arg-1 and low levels of CD86, CD80 and HLA-DR comparable to in vitro differentiated M2c macrophages. Represented is the geometrical mean for each target (target/isotype) with n = 5 experiments. (TIF) [file pone.0182039.s003.tif]

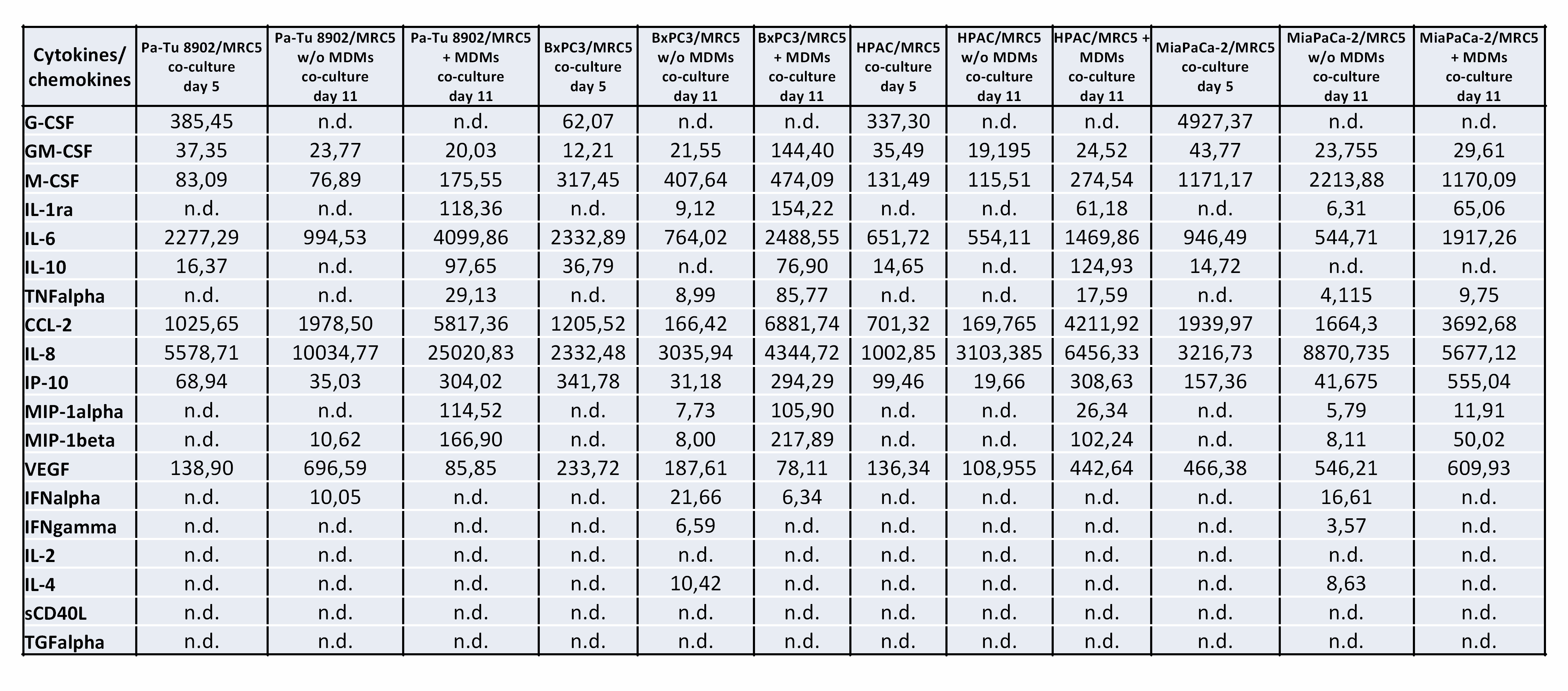

Supplement: S2 Table — Tumor cells and fibroblasts were co-cultured for 5 days. Monocytes were added to co-culture on day 5 and further cultivated for 6 days. Supernatants were collected on day 5 before monocyte addition and on day 11 from co-cultures without and with monocytes. A panel of 19 soluble factors was measured using Luminex multiplex technology or ELISA. Increased levels of several cytokines and chemokines could be detected on day 11 after addition of monocytes (n.d. = not detectable). Shown is the mean concentration in pg/ml of n = 3 independent experiments. (TIF) [file pone.0182039.s004.tif]
